# Sensitivity of PCR analysis (melting curve method) in diagnosis of Pulmonary Tuberculosis (PTB) based on Bronchoalveolar Lavage (BAL) by bronchoscope

**DOI:** 10.12669/pjms.38.5.5480

**Published:** 2022

**Authors:** Jun Wei, Jun Sun, Xuefen Shuai, Junqing Ren, Xuedong Chen

**Affiliations:** 1 Jun Wei, Department of Respiratory and Critical Medicine, Xuancheng People’s Hospital, Xuancheng 242000, Anhui, China; 2 Jun Sun, Department of Respiratory and Critical Medicine, Xuancheng People’s Hospital, Xuancheng 242000, Anhui, China; 3 Xuefen Shuai, Department of Respiratory and Critical Medicine, Xuancheng People’s Hospital, Xuancheng 242000, Anhui, China; 4 Junqing Ren, Department of Respiratory and Critical Medicine, Xuancheng People’s Hospital, Xuancheng 242000, Anhui, China; 5 Xuedong Chen, Department of Respiratory and Critical Medicine, Xuancheng People’s Hospital, Xuancheng 242000, Anhui, China

**Keywords:** Tuberculosis (TB), Bronchoscopy, Bronchoalveolar Lavage Fluid (BALF), Polymerase Chain Reaction (PCR)

## Abstract

**Objectives:**

To evaluate the sensitivity of the real-time PCR assay melting curve method in the diagnosis of pulmonary tuberculosis (PTB) by analyzing bronchoalveolar lavage fluid (BALF) obtained from bronchoscopy.

**Methods:**

A total of 214 PTB patients who were treated at Xuancheng People’s Hospital respiratory and infection department during January 2018 to January 2021 were included in this study. Bronchoscopic bronchoalveolar lavage fluid (BALF) examined by polymerase chain reaction (melting curve method), BALF smear, BALF culture, lipoarabinomannan (LAM) antigen test for diagnosis of tuberculosis (TB) (LAM-TB), acid-fast stain (AFS), and serum adenosine deaminase (ADA) test were conducted respectively to compare their positive predictive values (PPVs).

**Results:**

Of the 214 patients with confirmed PTB, 84.11% were BALF melting curve method positive, significantly higher than the positive results yielded by other PTB screening tests, i.e., LAM-TB (69.16%), AFS (51.87%), ADA (49.07%), BALF culture (62.15%), and BALF smear (41.12%) (p<0.05, respectively). The PPVs were increased to 92.06%, 93.93%, 92.99%, 95.79%, and 91.12% when BALF melting curve method was performed in combination with LAM-TB, AFS, ADA, BALF culture, and BALF smear, respectively, significantly higher than that produced by BALF melting curve method or the combined use of any two of the non-BALF melting curve method tests (p<0.05, respectively).

**Conclusion:**

BALF melting curve method is an ideal diagnostic approach to PTB, which is of a higher diagnostic value compared with LAM-TB, AFS, ADA, BALF culture, and BALF smear.

## INTRODUCTION

Pulmonary tuberculosis (PTB) is a contagious bacterial infection of the lungs caused by the bacterium Mycobacterium tuberculosis (M. tb), which spreads easily from an infected person to others by the airborne route.^1^,^2^ Constantly improved treatment options mean that most PTB patients can be cured with timely diagnosis and treatment.^3^ With laboratory (lab) tests being the key to PTB diagnosis^4^, it is essential to improve the diagnostic performance of PTB screening tests to achieve early diagnosis and effective prevention and control of the disease.^5^ Currently, laboratory tests such as sputum smear, sputum culture, sputum stain for M. tb (acid-fast stain, AFS), lipoarabinomannan (LAM) antigen test (LAM-TB), and serum adenosine deaminase (ADA) test are commonly used for the diagnosis of PTB but have low positive predictive values. Against this background, molecular biological testing has been increasingly discussed in the field of PTB prevention and control to optimize PTB pathogen detection and identification.^6^

This study was done to observe the sensitivity of BALF melting curve method in the diagnosis of pulmonary tuberculosis and explore the clinical application value of BALF melting curve method in the diagnosis of pulmonary tuberculosis, 214 patients with bronchoscopic alveolar lavage fluid (BALF) were detected by polymerase chain reaction (PCR) using fluorescence probe melting curve method and automatic medical PCR analysis system^7^,^8^ in this study.

## METHODS

A total of 214 PTB patients who were treated at Xuancheng People’s Hospital hospital between during January 2018 to January 2021 were included in this study. There were 97 males and 117 females, with the mean age of (36.54 ±13.27) years (range: 18-74). In terms of severity of lung involvement, one pulmonary lesion was found in 162 patients, while two or more were detected in the rest 62 patients. Clinical manifestations were cough and productive cough (n =208), fever (n =154), hemoptysis (n =61), and fatigue (n =184). Imaging features included fibrous streaks (n =127), cavities (n =113), multiple diffuse nodules (n =27), ground-glass opacities (n =22), and multi-segment effusion/consolidation (n =31).

### Ethical Approval:

The study was approved by the Institutional Ethics Committee of Xuancheng People’s Hospital on June 16, 2019(No.:2017020), and written informed consent was obtained from all participants.

### Inclusion criteria:

A patient was rendered eligible for this study if he/she:

- Met the diagnostic criteria for PTB;^9^
- Aged between 18 and 75;
- Agreed to receive bronchoscopic alveolar lavage fluid polymerase chain reaction (melting curve method) examination, BALF smear, BALF culture, LAM-TB, AFS, and ADA tests;
- Had knowledge of this study and agreed to sign an informed consent.

### Exclusion Criteria:

- Complications such as bronchogenic cancer, bronchial asthma, and chronic obstructive pulmonary disease;
- Incomplete test results;
- Confirmed mental disorders or cognitive impairment.

LAM-TB, AFS, and ADA were performed during the hospital stay, while screening for contraindications to flexible bronchoscopy was completed before routine bronchoscopy. Bronchoalveolar lavage (BAL) was carried out by locating the affected lobes and segments via bronchoscopy and chest imaging. Disposable cytology brushes, lavage equipment, and 100-150 mL of stroke-physiological saline solution (SPSS) at 37°C were used for BAL, where negative pressure (50-80 mmHg) was applied to collect 50-75 ml of BALF for smear preparation. Then smear of BALF, culture of Mycobacterium tuberculosis in BALF and polymerase chain reaction performed melting curve method were operated in strict accordance with the kit instructions. The melting curve analysis system and its kit were purchased from Xiamen Zhishan Biotechnology Co., Ltd.

### Statistical Analysis:

Data processing was conducted using SPSS23.0. Measurement data were expressed as “mean ± standard deviation (m±sd)”, and enumeration data was represented by rates or percentages. The comparison was examined by the χ2 test, with results at a level lower than 0.05 as statistically significant.

## RESULTS

 By comparing the positive results produced by different PTB screening tests, it was found that BALF melting curve method had a PPV (84.11%) higher than other diagnostic approaches, including LAM-TB (69.16%), AFS (51.87%), ADA (49.07%), BALF culture (62.15%), and BALF smear (41.12%) (p<0.05, respectively). Table-I.

**Table I:** PPV comparison among different PTB screening tests.

Lab Test	Total Cases (n)	Negative Cases (n)	Positive Cases (n)	PPV (%)
LAM-TB	214	66	148	69.16^*^
AFS	214	103	111	51.87^*^
ADA	214	54	160	74.76^*^
BALF culture	214	81	133	62.15^*^
BALF smear	214	126	88	41.12^*^
BALF melting curve method	214	34	180	84.11

***Note:***

* p<0.05 compared with BALF melting curve method.

PPVs were 92.06%, 93.93%, 92.99%, 95.79%, and 91.12% when BALF melting curve method was performed in combination with LAM-TB, AFS, ADA, BALF culture, and BALF smear, respectively, which were significantly higher than the PPVs of BALF melting curve method alone and the combined use of two non-BALF melting curve method tests (p<0.05, respectively). Table-II.

**Table II:** Comparison of diagnostic results by combined use of two PTB screening tests.

Lab Test	Add-on Test	Total Cases (n)	Negative Cases (n)	Positive Cases (n)	PPV (%)
BALF melting curve method	LAM-TB	214	17	197	92.06^*Δ^
	AFS	214	13	201	93.93^*Δ^
	ADA	214	24	199	92.99^*Δ^
	BALF culture	214	9	205	95.79^*Δ^
	BALF smear	214	19	195	91.12^*Δ^
LAM-TB	AFS	214	60	154	71.96
	ADA	214	37	177	82.71
	BALF culture	214	42	172	80.37
	BALF smear	214	51	163	76.17
AFS	ADA	214	41	173	80.81
	BALF culture	214	60	154	71.96
	BALF smear	214	73	141	65.89
ADA	BALF culture	214	38	176	82.24
	BALF smear	214	34	180	84.11
BALF culture	BALF smear	214	58	156	72.90

***Note:***

* p<0.05 compared with BALF melting curve method;

Δ p<0.05 compared with any two of the non-BALF melting curve method tests.

## DISCUSSION

PTB is the world’s leading infectious disease that threatens public health worldwide. China has approximately 1.3 million new PTB cases every year, which accounts for 14.3% of the world’s annual incidence, ranking second among the high PTB-burden countries. Although China has made impressive progresses in PTB prevention and control, it is still one of the countries heavily burdened with the disease and faced with the enormity of the nationwide TB epidemic.^10^ At present, clinical diagnosis of PTB mainly depends on imaging, lab tests and observation of clinical manifestations and treatment outcomes^11^, among which lab tests are the mainstay of final diagnosis and sputum smear and culture of M. tb as the “gold standard”.^12^ Although sputum smear is inexpensive and easy-to-administer as a PTB screening test, it has a relatively low PPV and is less sensitive to sputum culture, which is comparatively time-consuming and highly responsive to anti-TB treatment. Therefore, bacteriological examination of sputum is considered to have a low PPV in PTB diagnosis.^13^

LAM-TB relies on western blotting and monoclonal antibody detection to identify M. tb and 12 other pathogenic Mycobacterium spp. in patients with suspected PTB, which has a relatively high PPV by purification of TB-specific peptide antigens based on immunogenicity and serum M. tb-specific antibody profiles.^14^ In this study, LAM-TB had a PPV of 69.16%, basically consistent with the study by Wang YM et al.^15^, where the LAM-TB test results showed that 72.1% of the culture-positive patients and 45.6% of the culture-negative patients had PTB. ADA is an important hydrolase involved in purine nucleotide metabolism. In blood, ADA primarily exists in erythrocytes, granulocytes, and lymphocytes. The ADA level is associated with lymphocyte activation and differentiation. In PTB cases, the substantial increase in the ADA level is suggested to result from enhanced T cell-mediated immunity.^16^ Zhang C et al.^17^ reported that the serum ADA level in PTB patients was significantly higher than in healthy individuals [(37.35 ±4.68) U/L vs (12.11 ±3.23) U/L]. This study showed that the ADA test had a PPV of 74.72% in the diagnosis of PTB.

BALF from the affected bronchi is characterized by a relatively high bacterial concentration and a low risk of contamination.^18^ If timely delivered to laboratories, BALF smear and culture can produce PPVs higher than sputum smear and culture. BALF smear results can be obtained within the day of sampling, and yet the PPV is lower than BALF culture, which, however, needs more time to produce a higher PPV. To some extent, these screening tests may hinder the early diagnosis and treatment of PTB.^19^ PCR is a technique that amplifies DNA and RNA in vitro through DNA replication.^20^ PCR assays for M. tb detection have the following advantages: 1) bacteria at a concentration lower than the detectable levels of regular screening tests can be identified after DNA or RNA amplification, which is beneficial to the early diagnosis of PTB^21^; 2) PCR assays are more sensitive to PTB without cavitation compared with traditional approaches; 3) in addition to viable bacteria, dead bacteria without DNA degradation can also be amplified to identify lesions in the lungs and prevent missed or erroneous diagnosis;^22^ 4) PCR reagents work in parallel with anti-TB drugs to examine whether M. tb DNA has been completely degraded through quantitative determination. Fluorescence PCR probe melting curve method (melt Pro ® TB tuberculosis integrated detection system) has many detection sites, high sensitivity, strong specificity, and fast speed, the results of which can be obtained in 2-3 hours. With patients being able to receive their test results on the day of sampling, it can assist the early diagnosis and timely treatment of PTB. melting curve method assay of BALF is demonstrated to improve the PPV from 60-70% to a higher level in PTB diagnosis.^15^ In this study, BALF melting curve method has a PPV of 84.11%, outperforming LAM-TB, AFS, ADA, BALF culture, and BALF smear used for the diagnosis of PTB. Moreover, BALF melting curve method has a higher PPV with LAM-TB, AFS, ADA, BALF culture, or BALF smear as an add-on test (PPV =92.06%, 93.93%, 92.99%, 95.79%, and 91.12%, respectively), demonstrating a higher degree of sensitivity for PTB diagnosis compared with BALF melting curve method alone and the combined use of two non-BALF melting curve method tests.

### Limitations of the study:

It includes small sample are included in the study, short follow-up time, and failure to divide and study the post-operative pathological types, therapeutic effects and prognosis of patients in a more detailed manner due to small sample size. In view of this, proactive countermeasures will be taken in the future to carry out more comprehensive studies on such patients, so that more scientific data can be made available to our clinicians.

## CONCLUSION

This study demonstrated that BALF melting curve method is an effective PTB screening test of a diagnostic value higher than LAM-TB, AFS, ADA, BALF culture, and BALF smear. In addition, it can be used for the detection of rifampicin-resistant TB. Therefore, it is an ideal laboratory test for PTB diagnosis.

### Authors’ Contributions:

**JW & JS:** Designed this study,prepared this manuscript, are responsible and accountable for the accuracy or integrity of the work.

**XS & XC:** Collected and analyzed clinical data.

**JR:** Significantly revised this manuscript.

## References

[1] Pai M, Behr MA, Dowdy D, Dheda K, Divangahi M, Boehme CC (2016). Tuberculosis. Nat Rev Dis Primers.

[2] Glaziou P, Floyd K, Raviglione MC (2018). Global Epidemiology of Tuberculosis. Semin Respir Crit Care Med.

[3] Yadav R, Sharma N, Khaneja R, Agarwal P, Kanga A, Behera D (2017). Evaluation of the TB-LAMP assay for the rapid diagnosis of pulmonary tuberculosis in Northern India. Int J Tuberc Lung Dis.

[4] Hasan Z, Shakoor S, Arif F, Mehnaz A, Akber A, Haider M (2017). Evaluation of Xpert MTB/RIF testing for rapid diagnosis of childhood pulmonary tuberculosis in children by Xpert MTB/RIF testing of stool samples in a low resource setting. BMC Res Notes.

[5] Machuca I, Vidal E, de la Torre-Cisneros J, Rivero-Roman A (2018). Tuberculosis in immunosuppressed patients. Tuberculosis en pacientes inmunodeprimidos. Enferm Infecc Microbiol Clin (Engl Ed).

[6] Sah AK, Joshi B, Khadka DK, Gupta BP, Adhikari A, Singh SK (2017). Comparative Study of GeneXpert MTB/RIF Assay and Multiplex PCR Assay for Direct Detection of Mycobacterium tuberculosis in Suspected Pulmonary Tuberculosis Patients. Curr Microbiol.

[7] Mechal Y, Benaissa E, El Mrimar N, Benlahlou Y, Bssaibis F, Zegmout A (2019). Evaluation of GeneXpert MTB/RIF system performances in the diagnosis of extrapulmonary tuberculosis. BMC Infect Dis.

[8] Gharsalli H, Mlika M, Sahnoun I, Maalej S, Douik El Gharbi L (2018). The utility of bronchoalveolar lavage in the evaluation of interstitial lung diseases:A clinicopathological perspective. Semin Diagn Pathol.

[9] Jabri H, Lakhdar N, El Khattabi W, Afif H (2016). Les moyens diagnostiques de la tuberculose [Diagnostic means for tuberculosis]. Rev Pneumol Clin.

[10] Fatima S, Kumari A, Das G, Dwivedi VP (2020). Tuberculosis vaccine:A journey from BCG to present. Life Sci.

[11] Lyon SM, Rossman MD (2017). Pulmonary Tuberculosis. Microbiol Spectr.

[12] Gressens SB, Billard-Pomares T, Leboite H, Cruaud P, Bouchaud O, Carbonnelle E (2021). Pulmonary tuberculosis:Evaluation of current diagnostic strategy. Infect Dis Now.

[13] Meaza A, Kebede A, Yaregal Z, Dagne Z, Moga S, Yenew B (2017). Evaluation of genotype MTBDRplus VER 2.0 line probe assay for the detection of MDR-TB in smear positive and negative sputum samples. BMC Infect Dis.

[14] Shi SD, Hsueh PR, Yang PC, Chou CC (2020). Use of DosR Dormancy Antigens from Mycobacterium tuberculosis for Serodiagnosis of Active and Latent Tuberculosis. ACS Infect Dis.

[15] Wang YM, Zhuang H, Zhang HJ (2004). Diagnostic value of PPD, TB-AB and LAM detection for culture-negative pulmonary tuberculosis. J Con Ed Phys.

[16] Salmanzadeh S, Tavakkol H, Bavieh K, Alavi SM (2015). Diagnostic Value of Serum Adenosine Deaminase (ADA) Level for Pulmonary Tuberculosis. Jundishapur J Microbiol.

[17] Zhang C, Li J, Zhou X (2015). Serum ADA and CRP changes in patients with pulmonary tuberculosis. Chin J Clin Rat Drug Use.

[18] Lee SH, Sung JY, Yong D, Chun J, Kim SY, Song JH (2016). Characterization of microbiome in bronchoalveolar lavage fluid of patients with lung cancer comparing with benign mass like lesions. Lung Cancer.

[19] Peng J, Song J, Wang F, Zuo P, Lu Y, Liu W (2021). Harnessing Big Data to Optimize an Algorithm for Rapid Diagnosis of Pulmonary Tuberculosis in a Real-World Setting. Front Cell Infect Microbiol.

[20] Green MR, Sambrook J (2019). Polymerase Chain Reaction. Cold Spring Harb Protoc.

[21] Green MR, Sambrook J (2018). The Basic Polymerase Chain Reaction (PCR). Cold Spring Harb Protoc.

[22] Popper HH, Winter E, Hofler G (1994). DNA of Mycobacterium tuberculosis in formalin-fixed, paraffin-embedded tissue in tuberculosis and sarcoidosis detected by polymerase chain reaction. Am J Clin Pathol.

